# Association Between Improvement in Baseline Mood and Long-Term Use of a Mindfulness and Meditation App: Observational Study

**DOI:** 10.2196/12617

**Published:** 2019-05-08

**Authors:** Argus J Athanas, Jamison M McCorrison, Susan Smalley, Jamie Price, Jim Grady, Paul Wehner, Julie Campistron, Nicholas J Schork

**Affiliations:** 1 Department of Biomedical Informatics University of California San Diego San Diego, CA United States; 2 Department of Bioinformatics and Systems Biology University of California San Diego San Diego, CA United States; 3 J Craig Venter Institute San Diego, CA United States; 4 Department of Psychiatry University of California Los Angeles Los Angeles, CA United States; 5 Stop, Breathe & Think Los Angeles, CA United States; 6 Positive Place Inc The Villages, FL United States; 7 The Translational Genomics Research Institute (TGen) Department of Quantitative Medicine Phoenix, AZ United States; 8 The City of Hope/Translational Genomics Research Institute IMPACT Center Duarte, CA United States

**Keywords:** mental health, smartphone, emotional well-being, mindfulness

## Abstract

**Background:**

The use of smartphone apps to monitor and deliver health care guidance and interventions has received considerable attention recently, particularly with regard to behavioral disorders, stress relief, negative emotional state, and poor mood in general. Unfortunately, there is little research investigating the long-term and repeated effects of apps meant to impact mood and emotional state.

**Objective:**

We aimed to investigate the effects of both immediate point-of-intervention and long-term use (ie, at least 10 engagements) of a guided meditation and mindfulness smartphone app on users’ emotional states. Data were collected from users of a mobile phone app developed by the company Stop, Breathe & Think (SBT) for achieving emotional wellness. To explore the long-term effects, we assessed changes in the users’ basal emotional state before they completed an activity (eg, a guided meditation). We also assessed the immediate effects of the app on users’ emotional states from preactivity to postactivity.

**Methods:**

The SBT app collects information on the emotional state of the user before and after engagement in one or several mediation and mindfulness activities. These activities are recommended and provided by the app based on user input. We considered data on over 120,000 users of the app who collectively engaged in over 5.5 million sessions with the app during an approximate 2-year period. We focused our analysis on users who had at least 10 engagements with the app over an average of 6 months. We explored the changes in the emotional well-being of individuals with different emotional states at the time of their initial engagement with the app using mixed-effects models. In the process, we compared 2 different methods of classifying emotional states: (1) an expert-defined a priori mood classification and (2) an empirically driven cluster-based classification.

**Results:**

We found that among long-term users of the app, there was an association between the length of use and a positive change in basal emotional state (4% positive mood increase on a 2-point scale every 10 sessions). We also found that individuals who were anxious or depressed tended to have a favorable long-term emotional transition (eg, from a sad emotional state to a happier emotional state) after using the app for an extended period (the odds ratio for achieving a positive emotional state was 3.2 and 6.2 for anxious and depressed individuals, respectively, compared with users with fewer sessions).

**Conclusions:**

Our analyses provide evidence for an association between both immediate and long-term use of an app providing guided meditations and improvements in the emotional state.

## Introduction

### Background

Behavioral conditions, neuropsychiatric diseases, and poor general mental health are seen as major contributors to morbidity, mortality, and lost productivity on a global scale. However, these factors are often overlooked in discussions about the current state of health care, which tend to focus on physical well-being [1]. Many studies suggest that mental health can play a large role in physical health, recovery from disease, and ultimately productivity and, therefore, should receive greater attention [2-4]. Unfortunately, there are serious questions about how mental health can be promoted and, in instances when it is called for, how relevant interventions can be prescribed and deployed efficiently in a cost-effective manner [5-7]. This is especially true given the number of people who may actually benefit from such interventions [8]. In light of this, there is enthusiasm for the development of smartphone apps that can not only monitor an individual’s health—both physical and mental—but also deliver content designed to help coach them through difficult times or provide a needed intervention. In fact, many smartphone apps have been developed, or are under development, to aid in health care via, for example, image-based diagnostics, glucose monitoring for diabetes, and physical fitness promotion [9,10]. For mental health management and intervention, there is growing enthusiasm for the development of smartphone platforms that provide guidance on mindfulness and meditation as a way of relieving stress and promoting mental health and well-being. Many of the resulting platforms have been or are undergoing testing in clinical studies [11-15].

The use of mobile phone apps in combating or mediating behavioral conditions, stress, negative emotional states, and elevating mood is also consistent with directions that public health and regulatory officials are considering. In fact, evidence is mounting from clinical trials showing that smartphone apps can be effective in a variety of settings. Agencies such as the US Food and Drug Administration (FDA) have created, and in instances passed, legislation allowing the filing and approval of mobile health apps as approved health technologies on the same level as in vitro diagnostics and drugs. Pear Therapeutics was one of the first companies to have a smartphone app for addiction approved for use by the FDA in 2016 [16]. Many other commercial and academic groups are developing smartphone apps for a wide variety of conditions that go beyond the simple direct-to-consumer market by seeking regulatory approval for their use in clinical contexts [17-19]. Unfortunately, not enough time has elapsed since the introduction of smartphone-based intervention apps to provide insight into their long-term repeated effects as well as their effects in real-world settings (ie, outside of clinical trials) [20-22].

### Objectives

Stop, Breathe & Think (SBT) has developed a smartphone app that provides guided meditations and mindfulness activities to promote self-awareness coaching to interested users. As noted, mindfulness and meditation have been shown to improve affect and mood and promote healthy thought patterns [23,24]. The SBT app prompts users before and after they are guided through meditation and mindfulness activities to provide an emotional, mental, and physical *check-in*, thereby allowing an assessment of an individual user’s emotional state and mood pre- versus postactivity in real time. As repeated uses of the app by SBT users are archived, longitudinal information on its users with regard to their long-term engagement with the app is retained. This allows further analysis of the influence of repeated engagements with the app on an individual user’s basal mood over time in real-world settings. We pursued such an analysis using data from SBT users who had at least 10 engagements with the app. The SBT app allows users to choose from more than 100 unique emotions to reflect their emotional state at the time they use the app. These emotions cover a range of human emotions including anger, remorse, anxiety, calmness, and enthusiasm. Users are guided through meditations that they can choose from based on an algorithm developed by SBT. We focused our analyses on the *baseline* (or *basal*) emotional state of a user, before he or she engaged in a guided meditation or mindfulness activity and were primarily interested in the long-term and repeated use effects of the SBT app on this baseline emotional state. Essentially, we wanted to ask the question if the continued use of the app lifted the spirits of the user over time. We were particularly interested in users who tended to pick emotions associated with depression and anxiety when engaging with the app before meditating.

## Methods

### The Basic Stop, Breathe & Think App

The SBT app is a multiplatform (ie, iOS, Android, and Alexa) app designed to guide users through meditations and mindfulness activities to alleviate stress, anxiety, and depression and improve the sense of well-being. Upon opening the app, a user can participate in an optional 10-second reflection period. After this optional reflection period, users describe their current mood, emotional state, and physical health by choosing from a number of emotions; the SBT app then provides suggestions for specific meditation and mindfulness activities. The user can choose from among the suggested activities after being asked to endorse up to 5 different characterizations of their mood and emotional state. A user can choose not to provide any input regarding their mood, emotional state, and physical health and simply engage in an activity.

**Figure 1:**
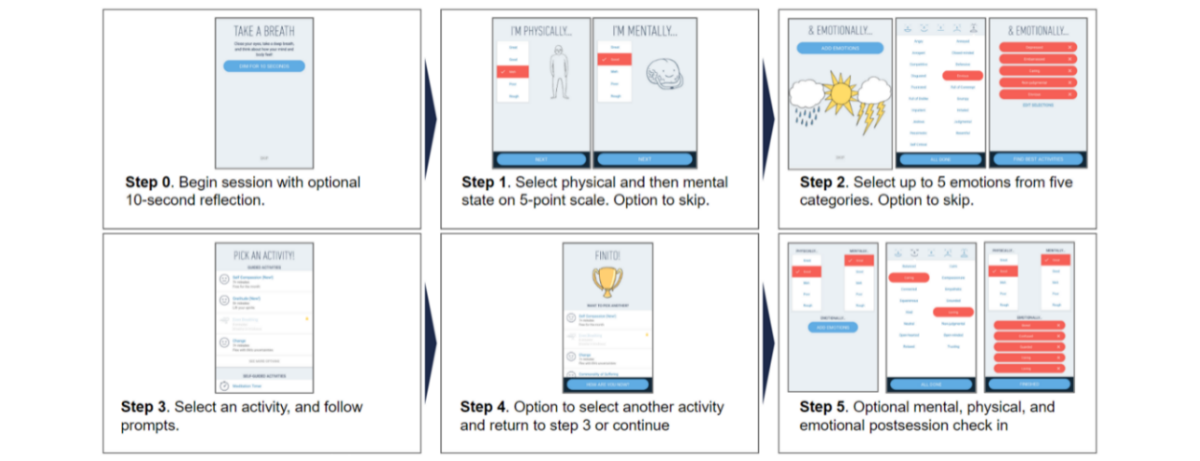
Stop, Breathe & Think user interface and stages of interaction with the app. Users are provided several ways in which they can record their current emotional state both pre- and postactivity. These emotional check-ins are optional, but the intuitive and simple selection process makes it easy for most users to enter at least some emotional status information.

Figure 1 provides a schematic of an individual session and the corresponding points where user information is collected.

It should be understood that all information collected with the SBT app is volunteered by users as stated and defined in the SBT user licensing agreement and privacy policy. In addition, for purposes of our data analyses, all the data we obtained from SBT were anonymized and put into a Health Insurance Portability and Accountability Act (HIPAA)-compliant format such that users could not be reidentified. Functionality and delivery of the SBT app and service varies from device and platform implementation (eg, Alexa, Android, and Web browser). Therefore, to avoid batch effects, we focused on users who were exclusively on an iOS platform and started using the app after SBT provided its last major version of the app (05/01/2016). Users had to have completed at least 10 sessions or engagements with the app, with a minimum of 6 of those sessions including pre- and postactivity emotion selections. The SBT app content is in English and to avoid translation errors and alternative interpretations of the language used in the SBT app, we restricted our analyses to individuals from native English-speaking countries: the United States, United Kingdom, Canada, and Australia. An additional filter was used, restricting users’ ages to between 12 and 100 years.

### Emotional Check-ins Pre- and Postactivity Score

The SBT app allows the user to endorse between 1 and 5 emotional states out of a possible 115, before and after engagement in a guided meditation or mindfulness activity (or series of activities if they choose to engage in more than 1 activity during a session). This emotional *check-in* involves selecting an initial emoticon and then choosing from a list of emotions within subgroups of terms that closest characterize the user’s current emotional state. These 115 emotions were chosen for the app based on internal SBT research and user requests. All emotions were classified as positive, neutral, or negative and given corresponding scores of 1, 0, and −1, respectively. All emotions and their corresponding scores are provided in Multimedia Appendix 1. As users can select up to 5 emotions, an average emotional score was calculated for both pre- and postactivity and standardized to a range from −1 (all negative emotions) to 1 (all positive emotions). Our analysis explored (1) trends in the preactivity emotional score over repeated uses of the app while accounting for the covariates as well as serial correlation between sessions and (2) trends in changes of the emotional scores before and after an activity over repeated uses of the app.

### Clustering of Emotions

In addition to treating the preactivity emotion scores and changes in emotion scores pre- and postactivity as dependent variables and time, sex, and age covariates as independent variables, we also explored the patterns among the emotion endorsements to see if there was evidence for obvious clusters of emotions that could reflect the same general emotional state. We leveraged principal coordinates analysis (PCoA) and the nonsupervised clustering technique, *Partitioning Around Medoids* (PAM), for these analyses [25]. We pursued these analyses as it is arguable that some users may see a subset of the emotions as synonymous and hence only choose one among many possible choices to describe their emotional state at the time to avoid redundancy, whereas other users might see those same subsets of emotions as complementary and reflecting different aspects of their mood. In addition, other users may preferentially select emotions based on their location in the selection list or choose a set of rare emotions that are infrequently selected by other users to differentiate their emotions.

The distance between the emotions was calculated using the Bray-Curtis distance measure [26]. To determine the optimal number of nonsupervised emotion clusters in 2-dimensional PCoA component space, we selected the number of clusters with the largest silhouette score. Once we identified the optimal number of clusters, emotions were then assigned to one of the identified clusters.

An individual’s emotional status was also summarized in terms of the relative *distances* (using the Euclidean distance measure) between pre- and postactivity states. The distances between an individual’s emotional status and the medoid of the closest associated emotion cluster were calculated as well. Emotions were labeled with clinical categories, associating each of them with either anxiety, depression, anger, or happiness (Multimedia Appendix 1). Ultimately, using distances between emotional states and emotional clusters allowed us to build models relating the number of times users engaged with the app to gross changes in emotional states defined by the emotion clusters.

### Statistical Analyses to Identify Long-Term Changes in Emotional State

To assess the effects of the continued use of the app on the preactivity emotional state, we used Linear Mixed-Effects (LME) models and Generalized Linear Models (GLMs) as implemented in the lme4 package in R [27]. These analysis techniques can accommodate serial correlations among emotions over time and also account for both fixed (eg, sex) and random effects (eg, variation in preactivity emotional state or the degree to which use of the app changed the preactivity emotional state over time). We pursued different analyses to evaluate changes in the preactivity emotional state over time, including a model that considered the effect of the emotional states possessed by individuals at their first engagement with the app. These analyses considered both the emotion scores as the dependent variables as well as the use of the emotions as defined by the cluster analysis clinical labels as dependent variables. We also tested the effect of repeated uses of the app on the change in the emotional state pre- to postactivity by treating the ratio of pre- to postemotion score as a dependent variable.

We included several covariates in our analyses and tested them for their effects on the emotional state: session index (ie, 1 as the first use and 2 as the second use—which captures the repeated use of the app), gender, age, country of origin, subscription status, and whether the user remained anonymous (ie, did not fill out information in his or her account—which may indicate a fake or disengaged user). As there is large variability in the number of completed sessions and the distribution of the number of uses of the app per individual has an extreme right skew, we applied a log_10_ transformation to the session index variable. This transformation markedly improved the normality of the session index as a variable (data not shown). LME models were fit, and the features associated with the preactivity emotional state as the dependent variable were selected using a forward stepwise selection procedure based on the Akaike Information Criteria. Similar models were fit with the pre- to postactivity emotional state ratio as the dependent variable. GLMs were fit to the data when changes in emotion categories (ie, based on clinical or cluster analysis labels) were taken as the dependent variable.

## Results

### Defining the Dataset

After all the duration, quality, platform, and country filters were applied, 13,393 users remained (10,082 females, 2187 males, and 1124 undeclared sex). The average age of the users was 32.3 (SD 13.5) years, with 31.7 (SD 13.3) years for females, 34.6 (SD 13.4) years for males, and 33.3 (SD 15.0) years for undeclared participants. Collectively, the users completed 569,961 sessions with the app, with 302,514 of these sessions having emotional check-in data, with an average of 42.6 sessions and 22.6 emotional check-ins per user. Multimedia Appendix 2 provides a histogram depicting the distribution of the length of time users engaged with the app. Multimedia Appendix 3 shows average period between app uses given the total length of engagement for users.

### Cluster Analysis of the Emotions

**Figure 2:**
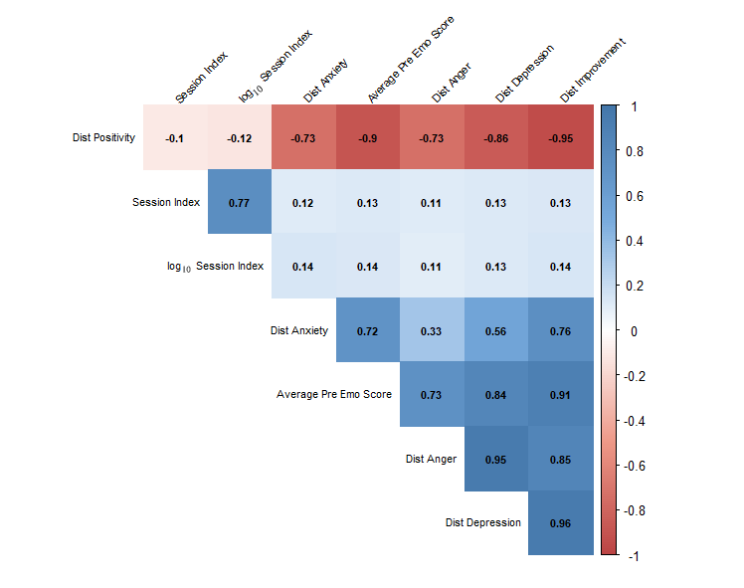
Average emotional score versus cluster centroid distances correlation matrix represented as a heat map. As an example for interpreting the numbers in the matrix, a −0.90 correlation between the preactivity emotion score (x-axis Average Pre Emo Score label) and positivity cluster (y-axis Dist positivity label) shows that users who score higher on the preactivity emotional score had a shorter distance of their selected emotions to the centroid of the positive emotion cluster. Note that labels with Dist reflect distance measures derived from the cluster analyses (eg, Dist Anxiety reflects the distance of a user’s emotional score from the anxiety cluster mean) and Emo reflects a specified emotional cluster.

**Figure 3:**
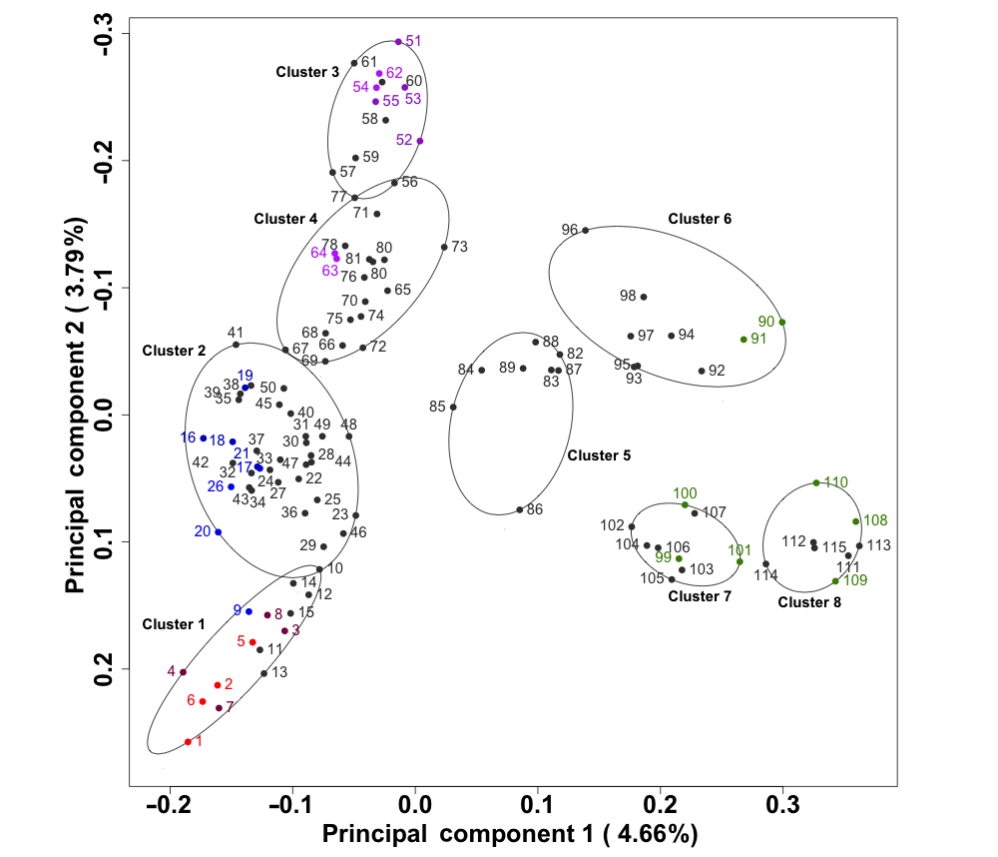
Emotion clustering using both pre- and postactivity emotion endorsements. The points in the plot reflect positions in the first 2 principal components defined by the Bray-Curtis distance between each pre- and postactivity emotional selection. The 8 circular clusters encompassing the emotions were defined by a permutation around medoids analysis technique, in which 8 clusters maximized the average cluster silhouette scores. Cluster boundaries are drawn on the smallest region including all underlying emotions. Emotions are labeled by clinical association such that terms clinically associated with anger are in red and pink, depression in blue, anxiety in purple, and happiness in green.

The use of the silhouette scores based on the PCoA and PAM analyses suggested that there were likely 8 clusters of emotions [28]. As noted, the relative distances between pre- and postactivity emotional states and the distances between each user’s emotional state and the closest associated emotion cluster were calculated. In addition, each of the 115 emotions that could be endorsed was assigned to one of the emotion clusters (see Multimedia Appendix 1). Using these cluster labels, we calculated the mean orientation of each cluster and the relative distance of each individual’s emotional scores both pre- and postactivity from these means. These distances were compared with the other emotion scores we calculated and were highly correlated with them (Figure 2). Figure 3 provides a graphical depiction of the results of the clustering using the first 2 principal coordinates obtained from our analyses.

### Mixed-Effects Modeling: Long-Term Use Effect on Preactivity Mood and Emotional State

**Figure 4:**
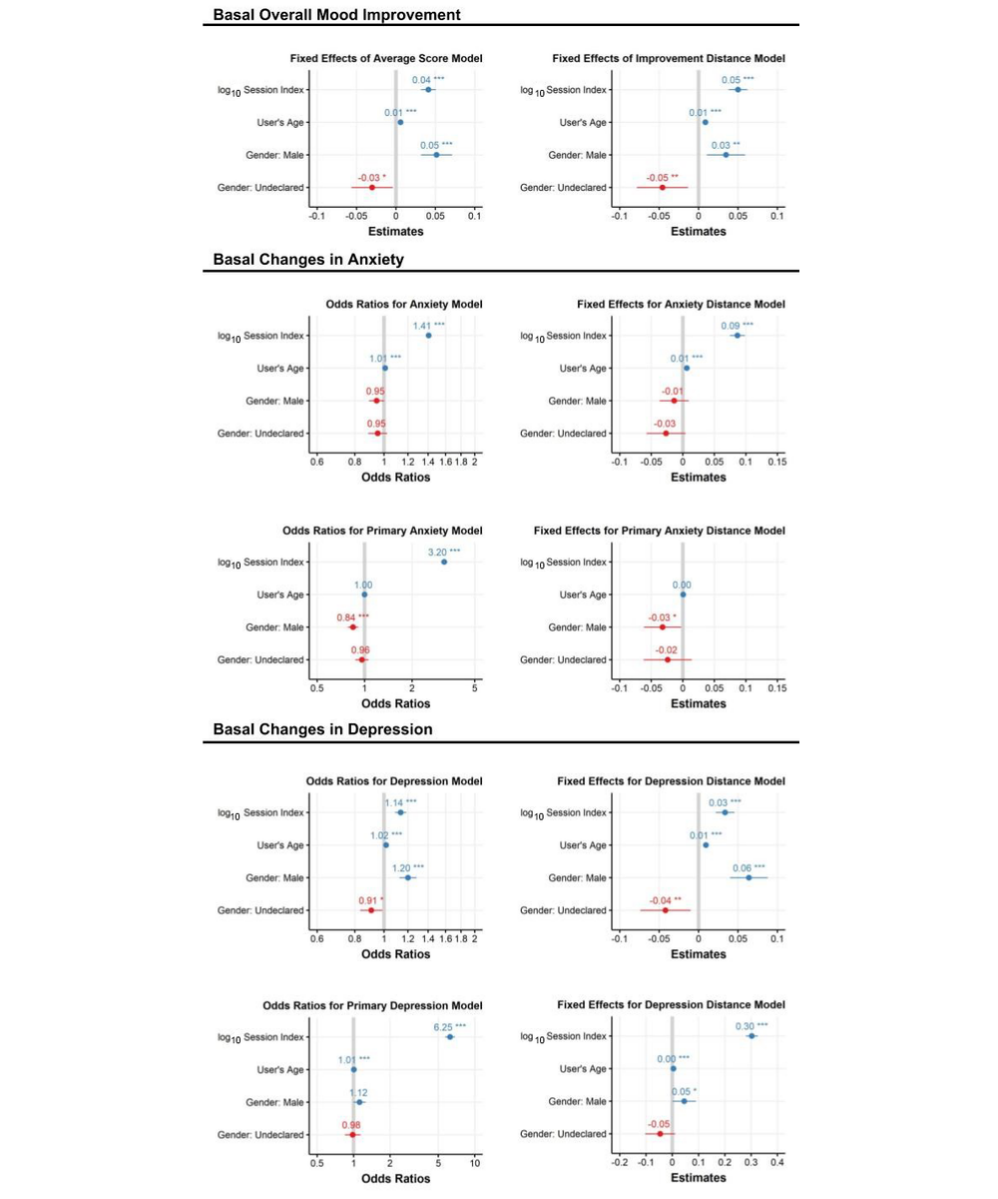
Linear mixed-effects regression coefficient estimates, their SEs, and P values (<.001***, <.01**, and <.05*) for models with the preactivity emotional state as the dependent variable. Analyses with the emotion scoring method as the dependent variable are on the left panels and analyses using distances from clustering as the dependent variable are on the right panels. Generalized Linear Model logit regression models were used with a binary dependent variable indicating if the emotion terms endorsed at a session reflected anxiety (middle panels) or reflected depression (bottom panels).

Using the average preactivity emotional scores, as well as the cluster-based distance measures, as dependent variables, we fit linear-mixed models with session, as well as the important covariates, as independent variables, while accommodating serial correlation emotions. The results using the average preactivity emotional state scores suggest that a statistically significant relationship exists between the number of uses of the app (ie, session index) and the preactivity emotional state, with an elevation in mood (ie, increase in positive emotions) occurring with repeated use of the app. Adjusting for scale, users experience a 2% improvement in mood after their first session, a 4% increase after their 10th session, and a 6% increase after their 100th session. The clinical relevance of this improvement in mood needs to be investigated further. We found that males have an average 2.5% higher (improved) preactivity mood than females and that older users have a more positive mood than younger users. Additional analyses suggested that repeated use of the app resulted in specific improvements in levels of anxiety and depression. After the first 10 sessions with the app—which on average corresponded to a 63.4-day period—users were 82% more likely to report no anxious emotions and 28% more likely to report no depressive emotions. This effect was even more pronounced when we only examined users whose first emotion endorsement reflected anxiety (440%) or depression (1050%). Figure 4 depicts the effect size and statistical significance of the estimated regression coefficients for the analysis models with the average emotional score in the left panels and cluster-based emotion similarity scores in the right panels. The statistical significance (ie, *P* values) were calculated using a Wald-Z statistic approximation. Models fit using a subset of users who reported anxious or depressed emotions in their first session with the app are labeled as *primary* models. The session index is consistently associated with improvements in mood, suggesting, again, that repeated use of the app positively impacts mood.

### Mixed-Effects Modeling: Pre- Versus Postactivity Mood or Emotional State

**Figure 5:**
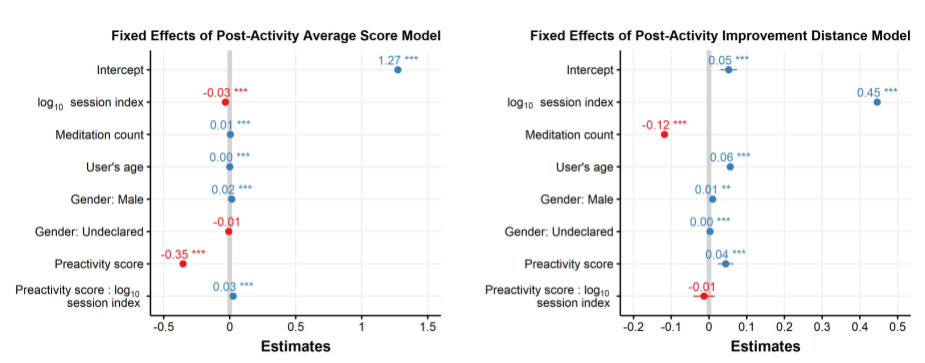
Linear mixed-effects regression coefficient estimates, their SEs and P values (<.001***, <.01**, and <.05*) for models with pre- to postactivity change in the emotional state as the dependent variable. An analysis with the standardized change in emotion score pre- to postactivity as the dependent variable is reflected in the top panel, and proximity to the positive emotional clusters as the dependent variable is reflected in the bottom panel.

We also fit models that considered the ratio of preactivity to postactivity emotional scores as the dependent variable. Figure 5 plots the regression coefficients resulting from the fits of these models with the ratio of average emotional score pre- to postactivity as the dependent variable (top panel) and the ratio of the distances between the emotions based on the clustering (bottom panel). The results suggest that repeated use of the app leads to increases in improvement of the mood/emotional state achieved through a meditation or mindfulness activity—or rather that the activities seem to lead to larger improvements in mood as the user has more engagements with the app.

## Discussion

### Principal Findings

Our analyses show that repeated engagements with the SBT app are associated with an improvement in users’ emotional states over time. In the absence of a randomized control trial, it is difficult to say with certainty that there is a direct causal relationship between the use of the SBT app and emotional state; however, given the large diverse sample size, we believe that the impact of unmeasured covariates on our results (such as external events in the users’ lives) is likely to be small, although potential biases in the users of the app may exist. The effect we observed is more pronounced for users who often endorse anxiety or depression when capturing their emotional state at their initial uses. We also found that age and sex covariates are associated with the basal mood or emotional state. Ultimately, our analyses suggest the possibility that guided meditations and mindfulness activities have the potential to be effective ways of reducing anxiety, depression, and stress and ultimately elevating mood, although the ultimate clinical significance of the improvements in the emotional state that we observed needs to be explored. Our analyses did reveal other interesting phenomena. For example, although a minority in our study, males tended to have higher baseline emotional scores and responded better to the SBT app than females. The age of a user was also found to be a significant correlate of the basal emotional state, with older users generally endorsing more positive emotions.

### Limitations of the Study

Our analyses are not without limitations, the first and foremost being that there is no control group and comparator app. This makes it difficult to definitively state that guided meditation and mindfulness activities are causally related or responsible for the increase in baseline mood or emotional state over time. However, given the sample size and magnitude of the effect, the significant change in emotional state after immediate and prolonged use of the app suggests that it has potential as an intervention. Another limitation is that all the information we analyzed was self-reported without any oversight by a third party. There could be users who did not follow instructions and entered erroneous emotions to expedite engagement with the meditations. Many of the individuals we did include in our analyses did not record emotions for each and every one of their sessions, resulting in many incomplete observations. Finally, a potential limitation with our analyses is that there could have been a heavy selection bias among the individuals using the app in the sense that they were motivated enough to download it and use it. Thus, this may be an indication that they could be predisposed to responding positively to the app.

### Broad Emotional State Transitions

Our use of the emotion clusters and similarity scoring of emotions based on our cluster analyses of those emotions allowed us to explore how often individual users transitioned from one broad set of analogous and almost synonymous emotions to another. On the basis of these analyses, we found evidence that, in general, individual users’ emotional states move from negative to positive over repeated uses of the app. We find that anxiety-prone and more depressed individuals benefit from the app more than others. These findings, as with the analyses, need to be verified in more controlled settings, such as randomized control trials, but again suggest that there is promise for the app and related apps in clinical and public health settings.

### Future Directions

There are a number of questions that deserve attention beyond those that we addressed with our data. For example, the number of uses of the app may not reflect the total length of time the app was used (eg, a user could engage with the app intensely over a short period of time or stretch their use out over a longer period of time). Assessing the impact of the number of uses versus length of time on outcomes could provide a more detailed insight into the benefits of the app. In addition, it would be good to see if a companion study designed especially for adolescent populations also has a positive effect on their emotions [29]. In addition, special clinical populations may benefit from the app (eg, clinically depressed individuals and individuals with addictions). It would be of value to explore analyses that focus on the impact of large-scale social stressors (eg, school shootings, national election results, and natural disasters) on the use of the app as well as its effects on mood in the wake of stress-inducing events. Geolocation data on users could better define such exposures to social stressors should they be location specific (eg, a natural disaster in a particular state). Finally, as emphasized, it would be ideal to test the utility of the app in bona fide clinical trials to determine which aspects of the app are causally related to improvements in mood and emotional state as well as identifying subgroups of individuals that appear to respond best to particular activities.

As more and more attention is given to the delivery of health care and health maintenance strategies through devices such as smartphones, robots, and telemedicine communications, greater sensitivity to the nuanced effects of these devices should motivate studies of them that are pursued in a comprehensive manner. Such sensitivity and more elaborate studies could also lead to more efficient and sophisticated deployment of these devices and help combat the need for expensive and logistically challenging visits to health care providers.

## Appendix

Multimedia Appendix 1 Assignment and scores for Stop, Breathe & Think selectable emotions.

Multimedia Appendix 2 Histogram of time from first to last recorded session for users with at least ten sessions and six emotional check-ins. On average users participated in sessions with the app over a period of 180 days, with a median use of 119 days, and maximum of 702 days.

Multimedia Appendix 3 Average user period between sessions. On average a user will interact with the app at least once every 6.34 days, and the majority of users complete at least two sessions per month.

## Abbreviations

FDA: Food and Drug Administration
GLM: Generalized Linear Model
LME: Linear Mixed-Effects
PAM: Partitioning Around Medoids
PCoA: Principal Coordinates Analysis
SBT: Stop, Breathe & Think
