# Correction: Association Between Improvement in Baseline Mood and Long-Term Use of a Mindfulness and Meditation App: Observational Study

**DOI:** 10.2196/28132

**Published:** 2021-06-30

**Authors:** Argus J Athanas, Jamison M McCorrison, Susan Smalley, Jamie Price, Jim Grady, Paul Wehner, Julie Campistron, Nicholas J Schork

**Affiliations:** 1 Department of Biomedial Informatics University of California San Diego San Diego, CA United States; 2 Department of Bioinformatics and Systems Biology University of California San Diego San Diego, CA United States; 3 J Craig Venter Institute San Diego, CA United States; 4 Department of Psychiatry University of California Los Angeles Los Angeles, CA United States; 5 Stop, Breathe & Think Los Angeles, CA United States; 6 Positive Place Inc The Villages, FL United States; 7 The Translational Genomics Research Institute (TGen) Department of Quantitative Medicine Phoenix, AZ United States; 8 The City of Hope/Translational Genomics Research Institute IMPACT Center Duarte, CA United States

In “Association Between Improvement in Baseline Mood and Long-Term Use of a Mindfulness and Meditation App: Observational Study” (JMIR Ment Health 2019;6(5):e12617) the authors noted one error.

In the originally published paper, author Paul Wehner's name was inadvertently not included in the list of authors, and the full list of authors and affiliations appeared as follows:

Argus J Athanas^1*^, BSc; Jamison M McCorrison^2,3*^, BSc; Susan Smalley^4^, PhD; Jamie Price^5^, JD; Jim Grady^5^, BA; Julie Campistron^5^, MBA; Nicholas J Schork^1,3,6,7^, PhD

^1^Department of Biomedical Informatics, University of California San Diego, San Diego, CA, United States

^2^Department of Bioinformatics and Systems Biology, University of California San Diego, San Diego, CA, United States

^3^J Craig Venter Institute, San Diego, CA, United States

^4^Department of Psychiatry, University of California Los Angeles, Los Angeles, CA, United States

^5^Stop, Breathe & Think, Los Angeles, CA, United States

^6^The Translational Genomics Research Institute (TGen), Department of Quantitative Medicine, Phoenix, AZ, United States

^7^The City of Hope/Translational Genomics Research Institute IMPACT Center, Duarte, CA, United States

^*^these authors contributed equally

The corrected list of authors and affiliations is as follows:

Argus J Athanas^1*^, BSc; Jamison M McCorrison^2,3*^, BSc; Susan Smalley^4^, PhD; Jamie Price^5^, JD; Jim Grady^5^, BA; Paul Wehner^6^, BA; Julie Campistron^5^, MBA; Nicholas J Schork^1,3,7,8^, PhD

^1^Department of Biomedical Informatics, University of California San Diego, San Diego, CA, United States

^2^Department of Bioinformatics and Systems Biology, University of California San Diego, San Diego, CA, United States

^3^J Craig Venter Institute, San Diego, CA, United States

^4^Department of Psychiatry, University of California Los Angeles, Los Angeles, CA, United States

^5^Stop, Breathe & Think, Los Angeles, CA, United States

^6^Positive Place Inc, The Villages, FL, United States

^7^The Translational Genomics Research Institute (TGen), Department of Quantitative Medicine, Phoenix, AZ, United States

^8^The City of Hope/Translational Genomics Research Institute IMPACT Center, Duarte, CA, United States

^*^these authors contributed equally

The correction will appear in the online version of the paper on the JMIR Publications website on June 30, 2021, together with the publication of this correction notice. Because this was made after submission to PubMed, PubMed Central, and other full-text repositories, the corrected article has also been resubmitted to those repositories.

