# Supplementary figures and images for: Association Between Improvement in Baseline Mood and Long-Term Use of a Mindfulness and Meditation App: Observational Study
Source: JMIR Ment Health. 2019 May 8;6(5):e12617. doi: 10.2196/12617 (PMC6707590; doi:10.2196/12617)

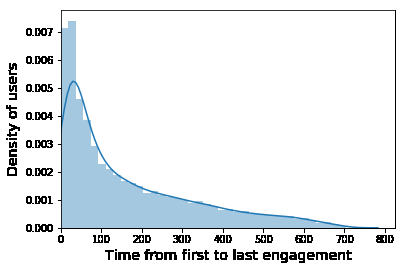

Supplement: Multimedia Appendix 2 [file mental_v6i5e12617_app2.png]

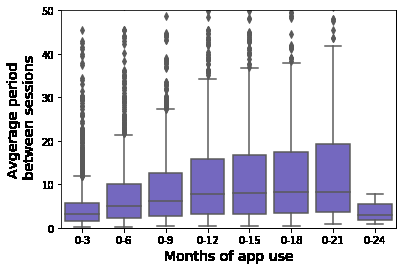

Supplement: Multimedia Appendix 3 [file mental_v6i5e12617_app3.png]
